# Correction to: Industrial antifoam agents impair ethanol fermentation and induce stress responses in yeast cells

**DOI:** 10.1007/s00253-017-8643-4

**Published:** 2017-11-25

**Authors:** Jens Christian Nielsen, Felipe Senne de Oliveira Lino, Thomas Gundelund Rasmussen, Jette Thykær, Christopher T. Workman, Thiago Olitta Basso

**Affiliations:** 1 Novozymes Latin America Ltda, Araucária, 83707-660 Brazil; 2 Department of Biotechnology and Biomedicine, Technical University of Denmark, DK2800 Kgs. Lyngby, Denmark; 3 Present Address: Department of Biology and Biological Engineering, Chalmers University of Technology, SE412 96 Gothenburg, Sweden; 4 Present Address: Department of Chemical Engineering, Polytechnic School, University of São Paulo, São Paulo, 05508-010 Brazil

**Correction to: Appl Microbiol Biotechnol (2017) 101:8237–8248**

10.1007/s00253-017-8548-2

The article **“Industrial antifoam agents impair ethanol fermentation and induce stress responses in yeast cells”**, written by Jens Christian Nielsen, Felipe Senne de Oliveira Lino, Thomas Gundelund Rasmussen, Jette Thykær, Christopher T. Workman and Thiago Olitta Basso, was originally published Online First without open access. After publication in volume 101, issue 22, page 8237–8248, the author decided to opt for Open Choice and to make the article an open access publication. Therefore, the copyright of the article has been changed to © The Author(s) 2017 and the article is forthwith distributed under the terms of the Creative Commons Attribution 4.0 International License (http://creativecommons.org/licenses/by/4.0/), which permits use, duplication, adaptation, distribution and reproduction in any medium or format, as long as you give appropriate credit to the original author(s) and the source, provide a link to the Creative Commons license, and indicate if changes were made.

The original article was corrected.

